# Patient and caregiver experiences of barriers to longitudinal care in coccidioidal meningitis in California’s central valley: a qualitative study

**DOI:** 10.1016/j.lana.2026.101513

**Published:** 2026-05-25

**Authors:** Geetha Sivasubramanian, Rayne Shepard, Uday Chauhan, John Christman, Nancy J. Burke

**Affiliations:** a Division of Infectious Diseases, Department of Medicine, University of California, San Francisco, Fresno, 155 N Fresno St, Fresno, CA 93701, USA; b Clinical Research Center, University of California, San Francisco, Fresno, 155 N Fresno St, Fresno, CA 93701, USA; c Public Health Department, University of California, Merced, 5200 N Lake Rd, Merced, CA 95343, USA

**Keywords:** Coccidioidal meningitis, Patient experience, Caregiver experience, Barriers to care, Endemic mycoses, Health equity

## Abstract

**Background:**

Coccidioidomycosis (Valley fever) is an endemic fungal infection of the southwestern United States, with an estimated 273,000 symptomatic infections, 23,000 hospitalizations, and 900 deaths annually. Coccidioidal meningitis, the most severe manifestation, requires lifelong antifungal therapy and carries high rates of rehospitalization, nonadherence, and mortality. Quantitative studies have documented poor outcomes, but the barriers driving these outcomes have not been examined from the patient’s perspective. This study aimed to characterize structural and systemic barriers to longitudinal care as experienced by patients with coccidioidal meningitis and their caregivers in California’s Central Valley.

**Methods:**

We conducted semi-structured interviews with patients with coccidioidal meningitis and caregivers at a tertiary referral center serving California’s Central Valley. Interviews explored pathways to diagnosis, treatment experiences, care coordination, and financial burden. Transcripts were analyzed using thematic analysis informed by the Consolidated Framework for Implementation Research.

**Findings:**

Ten patients and three caregivers participated. Participants were predominantly Hispanic and publicly insured. Five themes emerged: diagnostic delays, with patients requesting coccidioidomycosis testing themselves; gaps in patient awareness and provider communication of disease severity; fragmented care coordination; insurance barriers, including narrow specialist networks and pharmacy-level obstacles; and financial toxicity, with patients unable to work and forgoing care due to cost. Caregivers served as essential advocates, filling gaps in information retention and care coordination.

**Interpretation:**

Patients with coccidioidal meningitis described multiple barriers from initial presentation through ongoing management. These findings suggest that improving outcomes requires attention to the systems patients and their caregivers navigate after diagnosis, not solely to clinical management.

**Funding:**

University of California, San Francisco, Fresno, and Inspire Health Medical Group Intramural grant.

**Research in context**

**Evidence before this study**

We searched PubMed and Google Scholar from database inception through January 2025 using terms including “coccidioidomycosis,” “Valley fever,” “meningitis,” “patient experience,” “qualitative,” “barriers to care,” “adherence,” and “health disparities.” We included publications in English and Spanish. Quantitative studies have documented diagnostic delays, racial and socioeconomic disparities in incidence and outcomes, and high rates of medication nonadherence, rehospitalization, and mortality among patients with coccidioidal meningitis. Epidemiologic analyses have linked neighborhood disadvantage to hospitalization risk. One recent study used patient interviews to develop a patient-reported outcome measure for disseminated coccidioidomycosis, but no qualitative studies have examined the structural and systemic barriers patients encounter in managing their disease. The mechanisms connecting social context to poor outcomes have not been explored from the patient’s perspective.

**Added value of this study**

This study provides the first qualitative exploration of the barriers patients and caregivers encounter across the care continuum in coccidioidal meningitis. We identified five interconnected barriers: diagnostic delays requiring patient-initiated advocacy; gaps in patient awareness and provider communication of disease severity; fragmented care coordination with limited primary care access; insurance barriers, including restricted specialist networks; and financial toxicity affecting both patients and caregivers. These findings reveal how systemic failures compound across the care continuum and suggest why patients struggle to remain in care despite effective antifungal therapy.

**Implications of all the available evidence**

Improving outcomes for patients with coccidioidal meningitis will require interventions that address the systems patients and caregivers navigate after diagnosis, including standardized testing protocols, structured communication of severity, care coordination support, and attention to insurance and financial barriers. These findings likely apply to other forms of coccidioidomycosis requiring long-term management. More broadly, the barriers we identified reflect challenges faced by rural underserved populations managing chronic infectious diseases requiring long-term specialty care, including HIV, tuberculosis, and other endemic mycoses. The WHO’s 2022 recognition of fungal infections as a significant global health threat underscores the relevance of understanding patient-experienced barriers in these conditions.

## Introduction

In 2022, the World Health Organization released its first Fungal Priority Pathogens List, recognizing fungal infections as a significant global health threat, with over 6.5 million invasive infections and 3.8 million deaths annually.^1^^,^^2^ Coccidioidomycosis, commonly known as Valley fever, is an endemic fungal infection caused by inhalation of *Coccidioides* spores found in soils of the southwestern United States, northern Mexico, and parts of Central and South America.^3^ California’s Central Valley represents the epicenter of disease activity in the United States, with incidence increasing nearly 800% from 2000 to 2018.^4^ Recent modeling estimates that 273,000 symptomatic infections occur annually in the United States, 10 to 18 times higher than reported through national surveillance, resulting in approximately 23,000 hospitalizations and 900 deaths each year.^5^ The geographic range of *Coccidioides* is expanding beyond traditionally endemic areas. Within the United States, cases have been increasingly reported in Washington, Utah, and other states not historically considered endemic.^6^^,^^7^ Beyond the United States, coccidioidomycosis is endemic in Mexico, Central America, and South America, including Brazil, Argentina, Paraguay, Colombia, and Venezuela, where the true burden of disease remains largely unknown due to limited clinical awareness and restricted access to diagnostic testing.^8^^,^^9^ While most infections are self-limited, approximately 1–3% disseminate beyond the lungs, with coccidioidal meningitis representing the most severe manifestation, requiring lifelong antifungal therapy, and carrying substantial morbidity and mortality.^10^

The burden of coccidioidomycosis is not evenly distributed. Hospitalization rates are nearly twofold higher among African Americans compared to Whites, and more than twofold higher among those residing in large rural towns compared to urban areas.^11^ Mortality follows similar patterns.^12^ A case-control study found that annual income below $15,000 was an independent socioeconomic disadvantage that directly contributes to disease severity.^13^ A statewide California survey found that overall awareness of coccidioidomycosis was 42.4%, but was lowest among Hispanic persons (26.4%) and younger adults aged 18–44 years (32.9%), and only 3.5% of individuals with risk factors for severe disease knew they were at increased risk.^14^

Diagnostic delays compound these disparities. A recent study found that 87% of cases experienced delayed diagnosis, with 71% experiencing worsening of the disease attributable to the delay.^15^ Initial presentations occurred in primary care settings (56%), emergency departments (33%), and urgent care centers (11%), yet the diagnosis was missed. Detection is complicated by nonspecific symptoms indistinguishable from bacterial or viral pneumonia, limited clinician awareness, and barriers to healthcare access that disproportionately affect underserved populations.

Despite effective antifungal therapy, outcomes for patients with coccidioidal meningitis remain poor: in a 10-year retrospective analysis from our center, 43% were nonadherent to therapy, 59% required rehospitalization, and 23% died, suggesting that barriers beyond treatment efficacy drive poor outcomes.^16^ Yet the nature of these barriers, from the patient’s perspective, remains unexplored. Why do patients struggle to remain in care? What systemic failures prevent effective longitudinal management? How do insurance coverage, care coordination, and financial hardship interact to compound disease burden? These questions cannot be answered solely through retrospective chart review or administrative data analysis; to date, no qualitative research has explored the experiences of patients managing coccidioidomycosis.

We conducted a qualitative study using in-depth interviews with patients with coccidioidal meningitis and their caregivers at a tertiary referral center serving California’s Central Valley. We focused on patients with meningitis because this population faces the greatest complexity of care– lifelong therapy, frequent specialist visits, and surgical interventions. Our objective was to characterize the structural and systemic barriers patients encounter across the care continuum. By centering the lived experiences of a predominantly Latino, publicly insured population, this study aims to inform policy and practice interventions that can bridge the longitudinal care gap for coccidioidal meningitis and other forms of coccidioidomycosis requiring long-term management in endemic regions.

## Methods

### Study design and theoretical framework

This qualitative study used in-depth, semi-structured interviews to explore patients’ experiences managing coccidioidal meningitis. The study was guided by the Consolidated Framework for Implementation Research (CFIR), focusing on two domains: the inner setting (hospitals and clinics) and the individuals involved (patients and caregivers).^17^ CFIR was selected because its inner setting domain provides constructs for examining how healthcare delivery structures shape patient experiences, while the individuals domain captures the knowledge, beliefs, and self-efficacy that patients bring to managing their illness. Both domains point to relational and contextual factors that impact the effectiveness of interventions (e.g., care). The study was conducted at UCSF Fresno, Community Regional Medical Center (CRMC), and allied clinics in Fresno, California, the primary tertiary referral center for the Central San Joaquin Valley. This study is reported in accordance with the Consolidated Criteria for Reporting Qualitative Research (COREQ) checklist.^18^

### Participants and recruitment

Patients were eligible if they had a confirmed diagnosis of coccidioidal meningitis and were receiving care at the Infectious Diseases clinic, which provides outpatient follow-up for patients initially diagnosed and managed during hospitalization at the affiliated CRMC. Diagnosis was confirmed by positive coccidioidal serology, cerebrospinal fluid findings, cultures, or histopathology. Patients were recruited through direct invitations from research team members during scheduled clinic visits, using purposive sampling to capture diversity in time since diagnosis, treatment complexity (including single vs. multiple antifungal regimens and shunt placement), and insurance status. Caregivers were invited to participate alongside patients when present and willing; they were not administered a separate interview guide, but contributed to the discussion. The study was designed to include ten patient interviews, a sample size appropriate for in-depth qualitative analysis to generate detailed accounts of patient experiences. Sex was abstracted from medical records. The sample included seven male and three female patients. Given the small sample size and qualitative design, sex-based differences in experiences were not analyzed separately.

### Clinical data abstraction

Demographic and clinical characteristics were abstracted from medical records. Race and ethnicity were abstracted from medical records, where they had been recorded based on patient self-report.

### Patient interviews

Interviews were conducted in person by three research team members trained in qualitative interviewing techniques, provided by a co-investigator with expertise in qualitative health research in safety-net settings. Interviews followed a semi-structured guide developed by the research team and informed by CFIR constructs in the “individuals” domain. The guide addressed pathways to diagnosis, treatment experiences, provider communication, care coordination, and experiences with care discontinuity or medication nonadherence (Appendix 1 and 2). While the guide provided structure, interviewers used open-ended probes to allow participants to share experiences in their own words.

Interviews were conducted in English (n = 7) or Spanish (n = 3) based on patient preference. Three patients had family members or caregivers present who also contributed to the discussion. Interview duration ranged from 17 to 67 min (median 41 min, IQR 34–50). Participants provided verbal informed consent prior to participation; the interviewer documented consent at each interview. Interviews were audio-recorded and transcribed verbatim. Spanish-language interviews were transcribed in Spanish and subsequently translated into English by a native Spanish speaker with Tier 1 translation certification. Participants received a modest gift card after the interview in appreciation for their time. Transcripts were not returned to participants for member checking due to considerations of participant burden and varying literacy levels.

### Data analysis

Transcripts were analyzed using thematic analysis informed by CFIR constructs. Two team members read each transcript and discussed initial codes based on both a priori domains from the interview guide and emergent concepts identified in the data. Transcripts were subsequently coded using Atlas.ti (2023), which facilitated the addition of new codes and the collapse of redundant codes as appropriate.^19^ Themes were derived iteratively through a process of grouping related codes, identifying patterns across participants, and developing higher-order thematic categories. A matrix of themes and associated codes was developed for discussion, followed by a narrative grouping of quotes associated with each theme. Thematic saturation was assessed through ongoing team discussion during the coding process. No new thematic codes emerged in the final interviews, with later transcripts reinforcing patterns identified in earlier ones.

### Ethics approval

The CRMC Institutional Review Board approved the study (IRB number 2023075, approved 04/16/2025). Verbal informed consent was obtained from all participants prior to participation, and participants were provided with a patient information sheet.

### Role of funding source

This study was funded by the University of California, San Francisco, Fresno, and Inspire Health Medical Group Intramural grant. The funder had no role in study design, data collection, data analysis, data interpretation, report writing, or the decision to submit for publication.

## Results

### Participant characteristics

A total of ten patients with coccidioidal meningitis and three caregivers participated in interviews. Participants were predominantly Hispanic (9/10, 90%), publicly insured (9/10 Medi-Cal, 90%), and employed in agriculture or independent labor (Table 1). Median time since diagnosis was two years (range 0.5–13.5 years). At diagnosis, cerebrospinal fluid (CSF) analysis was available for nine patients and demonstrated elevated white blood cell counts (median 121.5 cells/μL, range 1–320), elevated protein levels (median 154 mg/dL, range 28 to >2000), and low glucose levels (median 30 mg/dL, range 10–120).

**Table 1:** Study patient characteristics (N = 10).

Characteristic	N (%)
Demographics	
Age, median [range], years	52 [20–68]
Male sex, n (%)	7 (70%)
White Hispanic, n (%)	9 (90%)
African American, n (%)	1 (10%)
Underlying medical conditions	
None, n (%)	5 (50%)
Diabetes mellitus, n (%)	3 (30%)
Sjögren’s syndrome, n (%)	1 (10%)
Pregnant at time of diagnosis, n (%)	1 (10%)
Occupational exposure history	
Yes, n (%)	4 (40%)
No known occupational exposure, n (%)	6 (60%)
Highest level of education	
No high school diploma, n (%)	1 (10%)
High school diploma, n (%)	3 (30%)
Some college, n (%)	1 (10%)
Bachelor’s degree, n (%)	1 (10%)
Unknown, n (%)	4 (40%)
Insurance status	
Medi-CAL, n (%)	9 (90%)
Medicare, n (%)	1 (10%)
Laboratory findings	
Serum complement fixation titers, median [range]	1:32 [negative–1:256]
Blood cultures positive for *Coccidioides* spp., n (%)	4/8 (50%)
Cerebrospinal fluid cultures positive, n (%)	0/9 (0%)
Cerebrospinal fluid *Coccidioides* polymerase chain reaction (PCR) positive, n (%)	2/8 (25%)
Cerebrospinal fluid complement fixation titer, median [range]	1:12 [1:4–1:1024] (n = 8)
Imaging and surgical history	
Hydrocephalus on imaging, n (%)	5 (50%)
Cerebrospinal fluid diverting shunt placement, n (%)	6 (60%)
Initial antifungal regimen	
Fluconazole, n (%)	9 (90%)
Fluconazole plus amphotericin B, n (%)	1 (10%)
Outcomes	
Number of re-hospitalizations, median [range]	2.5 [0–13]

Denominator reflects available data; cases where testing was not performed were excluded.

All patients were receiving antifungal therapy at the time of interview, with treatment duration ranging from 6 months to 12 years (Table 1). Several required dose escalations or switches to alternative agents during treatment. At the time of the interview, current regimens included fluconazole 800 mg daily (4/10), fluconazole 600 mg daily (1/10), voriconazole 300 mg daily (1/10), isavuconazole 372 mg daily (1/10), itraconazole 200 mg twice daily (1/10), and the investigational agent olorofim 60 mg twice daily (1/10). No patients received intrathecal therapy. Six patients (60%) developed disease-related complications, including stroke (n = 2), shunt malfunction (n = 2), vasculitis (n = 1), arachnoiditis (n = 1), seizures (n = 1), and persistent neurologic deficits (n = 1).

### Thematic findings

Five interconnected themes emerged from the interviews: (1) diagnostic delays and failures; (2) gaps in patient awareness and provider communication; (3) fragmented care; (4) insurance and access barriers; and (5) financial toxicity.

#### Diagnostic delays and failures

Patients described prolonged diagnostic journeys characterized by repeated healthcare encounters in which coccidioidomycosis was not considered. They reported that symptoms were frequently dismissed as viral illness and that diagnostic testing was limited to chest radiography without serologic evaluation. Patients cycled through urgent care clinics and emergency departments before receiving an accurate diagnosis, often only after they or their families specifically requested coccidioidomycosis testing.

“They said it was a virus, you know, they did chest x-rays and all that … And that’s how I ended up going there the first time.” (Participant 1)

Patients and their advocates, frustrated by a lack of answers, described requesting coccidioidomycosis testing themselves after multiple visits and unsuccessful tests for common viruses.

“So that last time when I got mad, I told them ‘Well what virus is it? Do some f-ing tests … do Valley fever, do something.’” (Participant 1)

“Every visit to the ER was just an X-ray. No blood work, nothing. Just an X-ray …” (Participant 9, caregiver)

Patients described being diagnosed with benign conditions, including “pregnancy fatigue” and asthma, which delayed recognition of coccidioidomycosis, in some cases for months. These diagnoses persisted even when patients questioned them.

“During my pregnancy, I kept getting fevers, and everything got worse, but no doctor could figure out what was wrong. They all said it was just pregnancy fatigue …”(Participant 10)

#### Patient awareness and provider communication

Patient awareness of coccidioidomycosis before diagnosis was highly inconsistent. While some had prior knowledge through family or colleagues, others had never heard of the disease or dismissed it as a minor illness that would only cause brief sickness.

“Well, I heard it from here and there, but I didn’t really pay attention to it. I thought it was just like, you know, like, oh, you’re sick for a week. You know, just like a little minor thing, but now I’m experiencing things … It’s not minor.” (Participant 3)

“I was shocked. Because I thought, you know, the only person I knew that had Valley fever is a cousin by marriage … And she said for her, she said it was picking cotton in the cotton fields, and I said, “well, that ain’t going to be me because I ain’t …” (Participant 5)

This misunderstanding extended to the perceived risk of exposure, with some believing it posed a threat only to agricultural workers.

Patients perceived that the life-altering severity of coccidioidal meningitis was not clearly communicated at the time of diagnosis. Some who initially minimized the illness expressed regret for not taking prescribed medication or symptoms seriously, realizing in hindsight that the infection had the potential to “take your life.”

“Because we knew of it and we were like ‘Oh okay’ you know? But when it happened, it’s like, no you can die. Like this will take your life. You know? And that’s when people are like … I just don’t think enough people are taking it as seriously as they should.” (Participant 1)

One patient reported receiving more prognostic information from a nurse than from the physician, leading to the perception that the physician had not fully conveyed the gravity of the diagnosis.

“Actually, I got more information out of a nurse than I did from doctors. They just said, ‘It looks like you have Valley fever.’ Then a nurse came in and I told her that, you know, I have Valley fever and she goes, ‘Oh, wow … Well, you’re going to notice a lot of weakening of your system, of your muscular system. You can live through it, you can be strong, but it is forever …” (Participant 8)

Across interviews, patients described limited recognition of coccidioidomycosis’s potential severity, both before and after diagnosis.

#### Fragmented care

Once patients received an accurate diagnosis, they faced numerous challenges managing their care. Patients described being told to follow up with primary care providers, but some lacked a primary care physician altogether.

“He was like ‘well you got to follow up with your primary.’ I didn’t even have a primary at the moment…“(Participant 1)

Others faced significant difficulty accessing care from their existing primary care physician, with scheduling delays of up to 6 months.

Patients perceived that initial treatment was inadequate, with symptoms progressing while awaiting specialty care referrals to process. Practical challenges compounded these difficulties. Patients described long hold times when calling clinic offices to schedule appointments, disconnected calls, and appointments scheduled months in advance.

“Sometimes they leave you on the line for a long time, you’re on hold for up to three hours sometimes, and they never answer, even if it says that they will. But sometimes they don’t and sometimes they hang up the call.” (Participant 10)

Patients reported challenges coordinating among the multiple providers involved in their care, including primary care physicians, infectious disease specialists, neurosurgeons, neurologists, pulmonologists, and nephrologists. Some reported receiving conflicting instructions, or no instructions, from different providers and feeling caught between care teams.

“I feel like I had two teams and with my doctor now, when I asked her, ‘what can I do about this and this?’ She said, ‘I don’t know. ‘I’m like, I gotta find me some help cause she’s telling me right there this is not her thing.’” (Participant 5)

Patients described uncertainty about which specialists to see and which medications to take, ultimately making their own decisions about how to navigate their care.

Patients also described instances in which test results were not communicated to the ordering or follow-up physician. Limited interpreter availability posed an additional barrier for Spanish-speaking patients.

“Sometimes they don’t have interpreters, because they’re busy or because they’re not available for it.” (Participant 6, caregiver)

Work schedules, especially for those with independent or delivery jobs, made it difficult to accommodate fixed appointment times.

#### Insurance and access barriers

A major structural barrier centered on insurance and the limited network of specialized providers. Patients on government-funded insurance described significant struggles, including providers ceasing to accept their insurance and being forced to switch primary care physicians to maintain access to specialists. Patients perceived that very few clinics in the Central Valley were equipped to treat their condition and accept their insurance.

“It wasn’t good because we used to go right here to the clinic. But then they said that they didn’t accept my insurance. So then we had to switch to another doctor but then the doctor wouldn’t be available because he had a lot of family emergency … And I guess that’s the only clinic right here in the Valley. Is either that one or all the way to San Jose …” (Participant 6, caregiver)

“So, we did have to make that very, very, very, hard decision to switch over and find her a new primary care physician just so that she can keep seeing these specialties.” (Participant 9, caregiver)

Referrals to appropriate care often took time. Patients reported delays and lapses in communication.

“But due to my insurance, it took a while to get here, probably over a month and a half. So meanwhile, the Valley fever was progressing.” (Participant 1)

Insurance-related barriers also affected timely access to diagnostics and medication. Patients reported lengthy waits for insurance authorization for imaging studies, with insurers requiring extensive justification for recurring procedures.

“We didn’t get approved really fast with certain things. Like last time we needed a CT scan, and they said they needed more details of why he needs a CT scan every 6 months … stuff like that took a while to get approved. Insurance need to know why we are getting that done.” (Participant 6, caregiver)

Transportation posed a significant barrier, with insurance often not covering rides, forcing patients or their families to pay out of pocket or rely on an ill person’s ability to drive.

“I’ve checked with my insurance and they don’t cover transportation. So …” (Participant 8)

“We would struggle a lot to get rides. Because I guess you could get a ride through insurance. But he’s not technically … How did they word it? That he could drive, just that he can’t drive right now.” (Participant 6, caregiver)

Beyond coverage, logistical hurdles at pharmacies, such as limited antifungal supply and disputes over refill timing, led to patients receiving insufficient quantities or running out of medication.

“They wouldn’t have it ….sometimes they didn’t have the amount that he needed. So sometimes they would just give me like 15 pills just to wait until we get the pills or else we will look for another pharmacy because the pharmacy never had fluconazole. It was like the hardest because it will always be out and then we never have the quantity …” (Participant 6, caregiver)

#### Financial toxicity

The illness created significant financial hardship, primarily by limiting or eliminating the ability to work. Patients reported extreme fatigue that prevented them from performing jobs, especially those in agriculture involving heat and light exposure, leading to job loss and transition to Medi-Cal and disability. For self-employed individuals, losing work meant months without income. The financial burden extended to family caregivers who took unpaid time off to provide support, placing stress on the household’s financial stability.

“So, then we switched her over to Medi-Cal because they did let us know now that she’s not going to be working, you know, so she qualifies for Medi-Cal … And then as far as, like, income wise, we were able to get her to apply for disability. But, right now, we’re kind of going through the financial burden that, you know, unfortunately, disability is a temporary thing … from July to now, she has had no income … So, that’s been a struggle. She can’t walk. She doesn’t have the energy to, you know, even like, stay awake for two hours without her feeling tired or weak or whatever the case is.” (Participant 9, caregiver)

“Well, it was with like a bit of … like fear, that you’re going to leave, that you haven’t finished, that you still have kids. There are bills and debts and the job, more than anything, the job is what worried me. Like, who’s going to work for me and all that?…” (Participant 7)

Patients struggled to navigate the administrative process of applying for social and financial assistance, such as disability or In–Home Supportive Services (IHSS). Patients described difficulty obtaining physician documentation of their condition’s severity, with some perceiving reluctance or judgment from providers when requesting disability paperwork.

“ … I asked the nurse if I could get disability help, and she looked at me with big, angry eyes. I told my family, who were helping me translate, that it was frustrating. My cousins told me that if you talk about disability, they don’t like to deal with you.” (Participant 10)

“So it’s hard to explain because you see him, he looks perfectly fine … So it’s like it’s hard to say that he can’t do certain things because then you see that he drives. So it’s like, how is it that possible if he could drive, but he can’t remember to take a pill or why is it possible that he could ride a bike, but he can’t jump or run. It doesn’t make sense, but it’s hard to say that he can’t do certain things because he looks perfectly fine.” (Participant 6, caregiver)

Some patients reported forgoing or delaying medical care due to financial strain. Before securing insurance, some described the costs as prohibitive, with fluconazole costing $250 for a two-week supply, leading them to stop attending appointments. Even with insurance, patients worried about medication costs, and families faced potential monthly costs of $7000 for a single medication if pre-authorization was denied.

“ … I worry a lot. I think, ‘I’m going to die,’ because I wouldn’t be able to pay for everything—like the bills, all the tests they do and everything.” (Participant 10)

“I don’t even know if that’s how expensive cancer medication is, I mean, I’m sure they’re expensive as well, but $7,000 a month? We don’t even make that income in a month. Hopefully, her insurance, Medi-Cal, approves this pre-authorization for Cresemba (isavuconazonium) …” (Participant 9, caregiver)

## Discussion

This study provides one of the first qualitative explorations of patient and caregiver experiences with coccidioidal meningitis. While Harvey et al. recently developed a patient-reported outcome measure for disseminated coccidioidomycosis through patient interviews, our study specifically examined the structural and systemic barriers patients encounter across the care continuum.^20^ Prior quantitative studies have documented poor outcomes in this population, including high rates of medication nonadherence, rehospitalization, and mortality, but why patients struggle to remain in care has not been examined from their perspective. Our interviews identified several barriers that may contribute to these outcomes. These barriers, while familiar in safety-net healthcare broadly, have not previously been documented from the patient perspective in coccidioidomycosis. Our five themes map onto CFIR’s inner setting and individuals’ domains, with diagnostic delays spanning inner and outer setting factors. While CFIR has been applied to HIV and tuberculosis program implementation, this study extends its use to understanding patient-experienced barriers in a chronic fungal infection requiring lifelong specialty care, an application that may inform similar analyses in other complex chronic conditions. This study prioritizes depth over breadth, drawing on detailed interviews with ten patients and three caregivers at a single center, and findings should be interpreted accordingly.

The clinical profiles of our participants reflect the complexity of coccidioidal meningitis management. The majority required ventriculoperitoneal shunt placement, and more than half developed disease-related complications requiring repeated hospitalizations and antifungal regimen changes. Managing this level of clinical complexity demands ongoing coordination among infectious disease specialists, neurosurgeons, and primary care providers, precisely the coordination that participants described as fragmented and difficult to navigate.

Several participants described diagnostic journeys in which coccidioidomycosis was not initially considered despite multiple healthcare encounters. In some cases, patients or family members reported requesting coccidioidomycosis testing themselves after repeated visits without a diagnosis. These experiences are consistent with quantitative studies documenting that the majority of patients with coccidioidomycosis are not tested during initial ambulatory visits, with diagnostic delays associated with substantial excess healthcare costs.^21^^,^^22^ While these quantitative studies have documented the frequency of diagnostic delays, our findings suggest that the experience of delay may involve active navigation by patients and families, rather than simply passive waiting. This raises questions about whether patients without informed advocates face additional challenges, though our study cannot address this directly.

Prior surveillance data have shown that awareness of coccidioidomycosis is low, particularly among Hispanic populations. Our findings suggest that awareness alone may not be sufficient; participants who had heard of coccidioidomycosis often dismissed it as minor. Several perceived that the seriousness of coccidioidal meningitis was not clearly communicated even after diagnosis. One participant reported learning that the infection was “forever” from a nurse rather than a physician. Another realized only later that it could “take your life.” These experiences raise questions about how severity and prognosis are communicated at the time of diagnosis. Structured frameworks such as the SPIKES (Setting, Perception, Invitation, Knowledge, Emotion, Strategy/Summary) protocol provide a stepwise approach to delivering serious diagnoses, but for chronic lifelong conditions, a single disclosure may be insufficient.^23^ Staged communication models, where prognostic understanding develops gradually across multiple consultations, have been recommended for conditions such as amyotrophic lateral sclerosis (ALS) and other serious chronic illnesses and may be applicable to coccidioidal meningitis.^24^

Participants described specific challenges coordinating care after diagnosis. Most patients with coccidioidal meningitis in the Central Valley are diagnosed during hospitalization, where they receive initial management from infectious disease specialists and, when needed, neurosurgeons. Upon discharge, patients require ongoing outpatient follow-up with these specialists for lifelong antifungal therapy, serologic monitoring, neuroimaging, and shunt management. However, transitioning from inpatient to outpatient specialty care requires referral through a primary care physician, who serves as the gatekeeper for specialist access under most California insurance plans, including Medi-Cal and many commercial HMO plans. Some participants were discharged with instructions to follow up with primary care providers they did not have; others faced months-long scheduling delays. The lack of primary care access described by participants, therefore, represents a structural barrier to continuing the specialty care initiated in the hospital. Recent studies at a California academic medical center found that the referral and authorization process in managed Medi-Cal drives patient care delays and fragmentation, resulting in difficulty accessing specialists, out-of-pocket costs, and disruptions to provider relationships due to frequent health plan changes.^25^^,^^26^

Some reported that test results did not reach their follow-up physicians. Interpreter services were inconsistently available. Participants who saw multiple specialists described receiving conflicting instructions and feeling uncertain about which providers to see. Practical barriers compounded these difficulties: long hold times, transportation not covered by insurance, and work schedules conflicting with appointments. These experiences suggest patients bear substantial responsibility for coordinating their own care, often without adequate support.

Insurance posed a distinct barrier for several participants. Those on government-funded insurance reported that providers had stopped accepting their coverage, forcing them to switch primary care physicians to maintain access to specialists. Participants perceived that very few clinics in the region could treat their condition and accept their insurance. Whether this reflects actual network limitations or difficulty navigating the system, the effect was similar: delays and uncertainty about where to go. Insurance authorization delays for imaging and medications, as well as pharmacy-level barriers, including limited antifungal supply and disputes over refill timing, further complicated treatment continuity.

Participants described substantial financial consequences from their illness. Several reported that fatigue prevented them from working, particularly in jobs that involved heat exposure, resulting in months without income. Recent data showing that symptoms such as fatigue, headache, and joint pain remain elevated up to 12 months after diagnosis further support the prolonged functional impact described by our participants.^27^ Disability benefits were described as temporary and insufficient. Caregivers reported taking unpaid leave to provide support and accompany patients to appointments. Some participants described forgoing appointments or medications due to cost. Patients who spend less on basic needs to afford medications have been shown to have worse adherence and health outcomes in other chronic diseases.^28^ These financial barriers are compounded by the communities where patients live. In California’s Central Valley, patients with coccidioidal meningitis are predominantly rural agricultural workers, and nearly all reside in areas with high Social Deprivation Index and low Healthy Places Index scores. Lower HPI scores have been associated with increased odds of hospitalization, suggesting that community-level disadvantage contributes to disease severity beyond individual clinical factors.^29^

Participants and caregivers described difficulty obtaining documentation of functional limitations because patients “look perfectly fine.” The mismatch between external appearance and internal impairment, including fatigue and cognitive difficulties, created challenges when applying for disability benefits and social services. Some perceived reluctance from providers when requesting disability paperwork. These experiences highlight the potential importance of accurate documentation of functional limitations for patients seeking support.

Caregivers played central roles in managing participants’ illness. They served as translators, advocates, navigators, and transportation providers. They managed medication regimens and absorbed the emotional weight of chronic illness. Patients recall less than half of the key information from medical consultations, and recall is worse with lower education and a higher information load.^30^ Transitional care interventions with caregiver engagement significantly reduce rehospitalizations, while those without do not.^31^ The burden of coccidioidal meningitis clearly extends beyond the patient to the household, and caregivers may have unmet needs of their own.

Several potential interventions merit consideration based on these findings. The diagnostic delays described by participants support consideration of standardized coccidioidomycosis testing protocols in emergency departments and urgent care centers in endemic regions. A dashboard-based system in Arizona that provides real-time coccidioidomycosis prevalence data to urgent care clinicians showed a strong correlation between testing frequency and public health case counts, suggesting that such systems could support earlier diagnosis.^32^^,^^33^ The experiences with severity communication suggest that how diagnoses are disclosed and whether patients understand the lifelong nature of their illness may warrant attention. Patient navigator programs have improved processes of care across chronic diseases in safety-net populations, including increased appointment adherence and reduced no-shows.^34^^,^^35^ Community health workers have demonstrated effectiveness in improving chronic disease control and reducing hospitalizations among low-income patients.^36^ Whether similar models would benefit patients with coccidioidomycosis warrants investigation.

This study has several limitations. Participants were recruited from a single tertiary referral center, which may not capture the experiences of patients who never reached specialty care. Our sample of ten patients, while appropriate for in-depth qualitative analysis, represents a small subset of those affected. The predominantly Hispanic, publicly insured population reflects Central Valley demographics but may limit transferability to other endemic regions. Additionally, although purposive sampling aimed to capture diversity in insurance status, all participants were publicly insured, reflecting the safety-net setting of our clinic. The experiences of commercially insured patients may differ and were not captured in this study. Treatment protocols varied across participants, including differences in antifungal regimens and surgical interventions, and experiences may differ based on treatment approach. All participants were infected with *C. immitis*, the species endemic to California. Although clinical behavior is not expected to differ substantially by species, the barriers patients face may differ in regions endemic for *C. posadasii* due to differences in healthcare infrastructure and access. Saturation assessment in small purposive samples is inherently informal, and we cannot exclude the possibility that additional interviews would have yielded new themes. As a qualitative study, we cannot quantify the prevalence of barriers or establish causal relationships. Interviews captured retrospective perceptions that may differ from how care was actually delivered. We did not systematically correlate individual patients’ reported barriers with their clinical trajectories, which would require a larger mixed-methods study. We focused on meningitis because these patients face the greatest complexity of care, but the barriers we identified, including insurance limitations, care coordination challenges, and financial hardship, are not specific to meningitis and likely affect patients with other forms of coccidioidomycosis requiring long-term management. Because participants were recruited from the clinic where they received care, social desirability bias may have limited critical discussion of experiences at the recruiting institution, even though interviewers were not involved in clinical care. Participants received a modest gift card after the interview in appreciation for their time, which may have influenced their willingness to participate in the study. Despite these limitations, the consistency of themes across participants supports the credibility of our findings.

The individual and structural challenges identified in this study, diagnostic delays requiring patient advocacy, fragmented care coordination, insurance network limitations, and financial toxicity, reflect realities faced by rural underserved populations managing chronic conditions more generally. While our focus on coccidioidal meningitis in California’s Central Valley provides a specific context, the barriers described are not unique to this disease or region. These findings have implications for chronic infectious diseases requiring long-term specialty care in underserved populations globally, including HIV, tuberculosis, and other endemic mycoses.

### Conclusion

This qualitative study identified barriers to care among patients with coccidioidal meningitis in California’s Central Valley, including diagnostic delays, inadequate communication of disease severity, fragmented care coordination, insurance limitations, and financial hardship. These barriers extended from initial presentation through ongoing disease management and affected both patients and their caregivers. The findings suggest that improving outcomes in this population will require attention to the systems patients navigate after diagnosis, rather than solely to diagnostic capacity. These lessons likely extend to other chronic conditions requiring long-term specialty care in underserved settings.

## Contributors

Geetha Sivasubramanian and Nancy Burke conceptualized, designed the study, and developed the interview guide. Nancy Burke provided training in qualitative interviewing techniques. Rayne Shepard and Uday Chauhan conducted the patient interviews, abstracted clinical data from medical records. John Christman and Nancy Burke coded and analyzed the transcripts. Geetha Sivasubramanian wrote the first draft of the manuscript. Geetha Sivasubramanian and Nancy Burke contributed to the interpretation of findings and critically revised the manuscript. All authors approved the final version. Geetha Sivasubramanian is the guarantor and accepts full responsibility for the work and has access to the data. Geetha Sivasubramanian, Rayne Shepard, and Uday Chauhan accessed and verified the underlying data. Geetha Sivasubramanian controlled the decision to publish.

## Data sharing statement

Deidentified interview transcripts and the interview guide will be made available to researchers who provide a methodologically sound proposal. Requests should be directed to the corresponding author. Data will be available beginning 12 months after publication, with no end date. A signed data access agreement will be required. The study protocol and coding framework will be available upon request. Individual participant clinical data will not be shared to protect participant confidentiality, given the small sample size and detailed narratives.

## Declaration of generative AI and AI-assisted technologies in the manuscript preparation process

During the preparation of this work, the author(s) used OpenEvidence (2025) to verify grammar, word count, and to follow formatting guidance. After using this tool/service, the author(s) reviewed and edited the content as needed and take(s) full responsibility for the content of the published article.

## Declaration of interests

All the authors have no financial interests to disclose.
