# Supplementary material for: Patient and caregiver experiences of barriers to longitudinal care in coccidioidal meningitis in California’s central valley: a qualitative study
Source: Lancet Reg Health Am. 2026 May 25;60:101513. doi: 10.1016/j.lana.2026.101513 (PMC13226943; doi:10.1016/j.lana.2026.101513)
Supplement: Appendix 1 [file mmc1.docx]

Cocci Study Interview

SCRIPT

*[Start recording]*

INTERVIEW QUESTIONS

1. General

I’d love to learn a little about you. Can you tell me about yourself?

- 1. How long have you lived in the area?
  2. What kind of work do you do?
  3. Can you tell me about your family?

1. Diagnosis
2. First, can you tell me about how you came to the clinic or hospital for Valley Fever?
   1. How did you get to the hospital? How long did it take?
   2. Would you mind walking me through how you were diagnosed?
      1. How many times did you go to the hospital for the same symptoms?
         1. How did you feel as things developed?
   3. How were you told about your illness?
      1. What kind of results were shared with you? How were they shared?
3. How did you feel at the time?
   1. Who was in the room when you had this conversation? Did anyone go with you to the hospital or clinic?
   2. Can you tell me about how it was explained to you?
   3. Who did you speak with about your diagnosis afterward?
4. Before being diagnosed, had you ever heard of Valley Fever?
   1. Can you talk a little about what you had heard about it?
      1. Did you know anyone who had it?
5. If you did your own research, where did you look?
   1. Can you tell me more about that?
   2. What happened next?
6. After understanding more about Valley Fever, how did your diagnosis make you feel?
7. [*If Spanish speaker]* Can you tell me about how this was communicated in Spanish? [probe for language ability of provider, or if interpreted, how]
   1. How did you feel about the interpretation? Can you talk about how that was arranged? [probe for information on work patient had to do to arrange this either through family or other]
8. Treatment understanding
9. Can you tell me what kind of treatment you are on?
   1. How as this explained to you?
   2. Can you talk a little about how you have been taking the medicine?
      1. Have you had to switch medications? If so, why?
   3. How does it make you feel?
   4. What kinds of challenges have you had with taking it?
      1. Insurance?
         1. What Insurance do you have?
      2. Side effects?
         1. What side effects?
   5. How has the treatment you are on affected your life?
   6. What kinds of conversations have you had with people from a clinic, primary care physician office, or hospital about your diagnosis or treatment?
      1. What kinds of questions have you [or another person] asked?
      2. Who? What did they say? How helpful was this?
      3. If you can think of a time when you were confused or weren’t quite sure what the provider was telling you about your health or treatment, please describe what happened?
10. Has there ever been a time when you stopped taking your medication and/or attending clinic appointments? Can you tell me about this?
11. Besides taking the medication, what other things do you do to make yourself feel better?
    1. How often do you do this?
    2. How does it make you feel?
12. [*If Spanish*] Can you tell me about how this was communicated in Spanish>? [probe for language ability of provider, or if interpreted, how?]
    1. How did you feel about the interpretation?
13. Experiences with healthcare, work, insurance, etc.
14. How did you end up coming to the Infectious Disease clinic at FHCN?
    1. What other places did you try first?
    2. How do you know when you have appointments?
       1. Has there ever been a time where it was difficult to make an appointment with this clinic?
          1. Do you utilize the MyChart app to keep track of your healthcare? How do you use it?
       2. Have you ever had trouble reaching someone at the clinic?
       3. Who do you usually talk to at the clinic?
          1. *[If Spanish]: How are these conversations interpreted?*
             1. *How do you feel about the interpretations?*
    3. Can you talk about your relationship with your doctor?
       1. How do you feel about the care you receive? Please feel free to be as honest as possible. I am not affiliated with this clinic.
          1. *[If Spanish]: How are these conversations interpreted?*
             1. *How do you feel about the interpretation?*
    4. How far do you travel to attend clinic appointments?
       1. How does this affect your life?
    5. How do you feel you contracted Valley Fever? Can you elaborate?
    6. How did this make you feel?
    7. What did you have to do to resolve these issues?
15. Have you encountered any issues with your health insurance?
    1. What happened?
    2. What insurance do you have?
    3. What did you have to do to resolve these issues?
    4. How did this make you feel?
16. Were there any other issues you encountered? Who helped you with these issues?
    1. Disability paperwork?
    2. Hospitalizations?
    3. Family obligations?
    4. Childcare?
17. Is there anything else you would like to add?

*Thank you so much for speaking with me today. I’ve learned so much from this conversation.*

*[Provide compensation]*

*[End recording]*
