# Supplementary material for: Patient and caregiver experiences of barriers to longitudinal care in coccidioidal meningitis in California’s central valley: a qualitative study
Source: Lancet Reg Health Am. 2026 May 25;60:101513. doi: 10.1016/j.lana.2026.101513 (PMC13226943; doi:10.1016/j.lana.2026.101513)
Supplement: Appendix 2 [file mmc2.docx]

Cocci Study Interview

SCRIPT

1. General - (5-7 minutos)

Me encantaría saber un poco sobre ti. ¿Puedes contarme algo sobre ti?

1. ¿Cuánto tiempo llevas viviendo en la zona?
2. ¿Qué tipo de trabajo haces?
3. ¿Puedes contarme algo sobre tu familia?
4. Diagnóstico (15 minutos)
5. Primero, ¿puedes contarme cómo llegaste a la clínica o al hospital por la fiebre del valle?
   1. ¿Cómo llegaste al hospital? ¿Cuánto tiempo te llevó?
   2. ¿Te importaría explicarme cómo te diagnosticaron?
      1. ¿Cuántas veces fuiste al hospital por los mismos síntomas?
         1. ¿Cómo te sentiste a medida que se desarrollaban las cosas?
   3. ¿Cómo te informaron sobre tu enfermedad?
      1. ¿Qué tipo de resultados te dieron a conocer? ¿Cómo te los dieron a conocer?
6. ¿Cómo te sentiste en ese momento?
   1. ¿Quién estaba en la habitación cuando tuviste esta conversación? ¿Alguien te acompañó al hospital o la clínica?
   2. ¿Puedes contarme cómo te lo explicaron?
   3. ¿Con quién hablaste sobre tu diagnóstico después?
7. Antes de que te diagnosticaran, ¿habías oído hablar de la fiebre del valle?
   1. ¿Puedes hablar un poco sobre lo que habías oído al respecto?
      1. ¿Conocías a alguien que la tuviera?
8. Si investigaste por tu cuenta, ¿dónde buscaste?
   1. ¿Puedes contarme más sobre eso?
   2. ¿Qué pasó después?
9. Después de entender más sobre la fiebre del valle, ¿cómo te hizo sentir tu diagnóstico?
10. [Si hablas español] ¿Puedes contarme cómo se comunicó esto en español? [Pregunta sobre la capacidad lingüística del proveedor o, si se trata de un intérprete, cómo]
    1. ¿Cómo te sentiste con la interpretación? ¿Puedes hablar sobre cómo se organizó? [Pregunta sobre el trabajo que tuvo que hacer el paciente para organizar esto, ya sea a través de la familia u otros]
11. Treatment understanding
12. ¿Puede decirme qué tipo de tratamiento está recibiendo?
    1. ¿Cómo se lo explicó?
    2. ¿Puede hablar un poco sobre cómo ha estado tomando la medicación?
       1. ¿Ha tenido que cambiar de medicación? Si es así, ¿por qué?
    3. ¿Cómo se siente?
    4. ¿Qué tipo de desafíos ha tenido al tomarla?
       1. ¿Seguro?
          1. ¿Qué seguro tiene?
       2. ¿Efectos secundarios?
          1. ¿Qué efectos secundarios?
    5. ¿Cómo ha afectado su vida el tratamiento que está recibiendo?
    6. ¿Qué tipo de conversaciones ha tenido con personas de una clínica, consultorio médico de atención primaria u hospital sobre su diagnóstico o tratamiento?
       1. ¿Qué tipo de preguntas ha hecho usted [u otra persona]?
       2. ¿Quién? ¿Qué le dijeron? ¿Qué tan útil fue esto?
       3. Si puede pensar en un momento en el que se sintió confundido o no estaba muy seguro de lo que el proveedor le estaba diciendo sobre su salud o tratamiento, describa lo que sucedió.
13. ¿Ha habido alguna vez en que dejó de tomar su medicación y/o de asistir a las citas clínicas? ¿Puedes contarme sobre esto?
14. Además de tomar la medicación, ¿qué otras cosas haces para sentirte mejor?
    1. ¿Con qué frecuencia haces esto?
    2. ¿Cómo te hace sentir?
15. [Si es español] ¿Puedes contarme cómo se comunicó esto en español? [Pregunta sobre la capacidad lingüística del proveedor o, si es interpretado, ¿cómo?]
    1. ¿Cómo te sentiste con la interpretación?
16. Experiences with healthcare, work, insurance, etc.
17. ¿Cómo llegó a la clínica de enfermedades infecciosas de FHCN?
    1. ¿Qué otros lugares probó primero?
    2. ¿Cómo sabe cuándo tiene citas?
       1. ¿Ha habido alguna vez un momento en el que le resultó difícil concertar una cita en esta clínica?
          1. ¿Utiliza la aplicación MyChart para realizar un seguimiento de su atención médica? ¿Cómo la utiliza?
       2. ¿Alguna vez ha tenido problemas para comunicarse con alguien en la clínica?
       3. ¿Con quién suele hablar en la clínica?
          1. [Si es español]: ¿Cómo se interpretan estas conversaciones?
             1. ¿Qué opina sobre las interpretaciones?
    3. ¿Puede hablar sobre su relación con su médico?
       1. ¿Cómo se siente sobre la atención que recibe? No dude en ser lo más honesto posible. No estoy afiliado a esta clínica.
          1. [Si es español]: ¿Cómo se interpretan estas conversaciones?
             1. ¿Qué opina sobre la interpretación?
    4. ¿Qué tan lejos viaja para asistir a las citas en la clínica?
       1. ¿Cómo afecta esto a su vida?
    5. ¿Cómo se siente al contraer la Fiebre del Valle? ¿Puede explicarlo con más detalle?
    6. ¿Cómo se sintió?
    7. ¿Qué tuvo que hacer para resolver estos problemas?
18. ¿Ha tenido algún problema con su seguro médico?
    1. ¿Qué sucedió?
    2. ¿Qué seguro tiene?
    3. ¿Qué tuvo que hacer para resolver estos problemas?
    4. ¿Cómo se sintió?
19. ¿Hubo otros problemas que tuvo? ¿Quién le ayudó con estos problemas?
    1. ¿Trámites de discapacidad?
    2. ¿Hospitalizaciones?
    3. ¿Obligaciones familiares?
    4. ¿Cuidado de niños?
20. ¿Hay algo más que le gustaría agregar?

*Muchas gracias por hablar conmigo hoy. He aprendido mucho de esta conversación.*

*[End recording.]*
